# Case report: Inflammatory sternoclavicular joint arthritis induced by an immune checkpoint inhibitor with remarkable responsiveness to infliximab

**DOI:** 10.3389/fimmu.2024.1400097

**Published:** 2024-05-10

**Authors:** Shion Kachi, Shuji Sumitomo, Hideki Oka, Akito Hata, Koichiro Ohmura

**Affiliations:** ^1^ Department of Rheumatology, Kobe City Medical Center General Hospital, Kobe, Hyogo, Japan; ^2^ Department of Thoracic Oncology, Kobe Minimally Invasive Cancer Center, Kobe, Hyogo, Japan

**Keywords:** irAE, immune checkpoint inhibitor (ICI), sternoclavicular arthritis, infliximab, durvalumab, programmed-death ligand 1 (PD-L1), small cell lung carcinoma (SCLC)

## Abstract

This report describes the case of a 48-year-old woman who presented with sternoclavicular joint arthritis after administration of an immune checkpoint inhibitor (ICI), durvalumab, for small cell lung carcinoma. The onset of arthritis transpired 18 months after the commencement of the ICI therapeutic regimen and demonstrated resilience to glucocorticoid treatment. After excluding infectious aetiologies and metastatic involvement, the patient was diagnosed with ICI-induced arthritis (ICI-IA). Considering the articular implications akin to the SAPHO syndrome, the patient was treated with infliximab, resulting in complete resolution. This finding implies that biological DMARDs can serve as effective interventions for ICI-induced sternoclavicular joint arthritis. Given the heterogeneous nature of its pathogenesis, the selection of therapeutic agents may require customization based on the distinct clinical presentation of each individual case.

## Introduction

The use of immune checkpoint inhibitors (ICIs) such as anti-programmed cell death protein 1 (PD-1)/programmed cell death-ligand 1 (PD-L1) and cytotoxic T-lymphocyte associated antigen 4 (CTLA-4) has greatly advanced the treatment of several types of cancer, including various solid-organ and hematologic malignancies (1). However, enhancing anti-tumor T-cell activity can lead to immune-related adverse events (irAEs), which are distinct from conventional treatments like chemotherapy (2). Most frequently affected organs are the skin, gastrointestinal tract, endocrine glands, and lung. Rheumatic and musculoskeletal irAEs, symptoms vary widely, with arthralgias and myalgias being frequently reported, occurring in 1–43% and 2–21% of patients, respectively (3, 4). Based on case series and case reports, autoantibodies are often absent, and around 20% of rheumatic irAEs patients fulfilled the classification criteria of rheumatoid arthritis or polymyalgia rheumatica (5). While joint symptoms often involve the shoulders, metacarpophalangeal, and proximal interphalangeal joints of the hands, axial joint involvement has been less frequently documented (5, 6). In this report, we present a case of sternoclavicular joint (SCJ) arthritis developing in a patient undergoing PD-L1 inhibitor, durvalumab, for over a year.

## Case description

**Figure 1:**
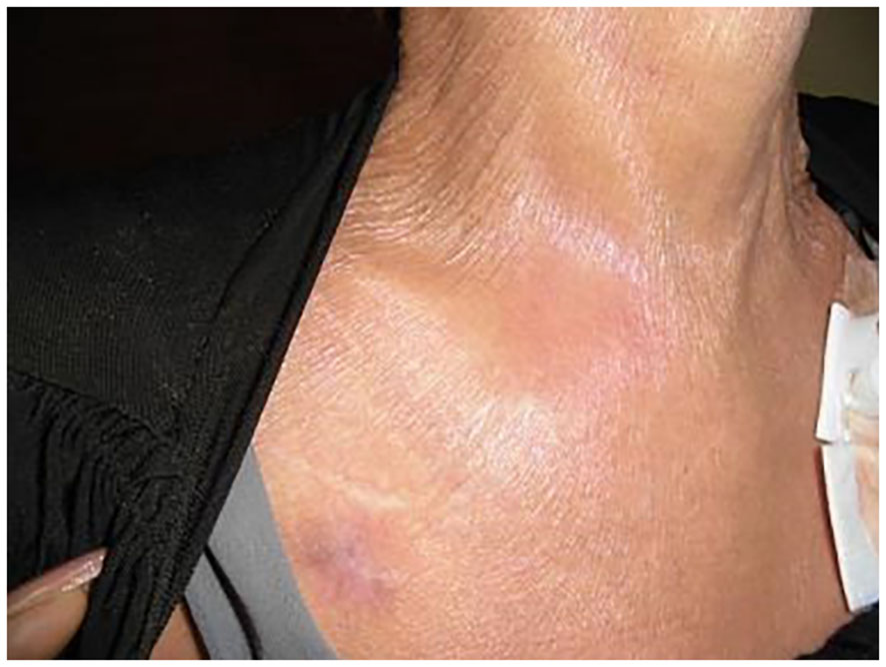
Erythematous swelling at the right sternoclavicular joint.

**Figure 2:**
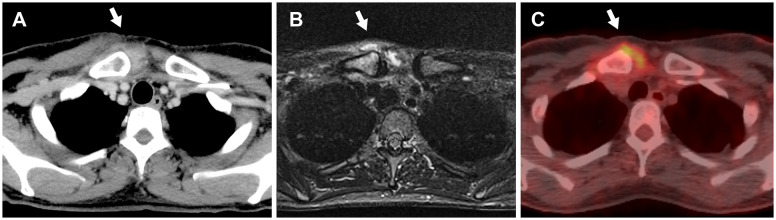
The imaging findings of the right sternoclavicular joint without evidence of bone fractures, abscesses, osteonecrosis, or metastasis (white arrows). **(A)** Computed tomography image shows increased fatty tissue concentration. **(B)** Magnetic resonance imaging with short tau inversion recovery reveals hyperintensities. **(C)** ^18^F-Fluorodeoxyglucose-positron emission tomography image demonstrates abnormal uptake (SUVmax = 4.1).

A 48-year-old woman underwent treatment for a stage IV small cell lung carcinoma with brain, pancreatic, and liver metastases. At the referring hospital, she received a combination of durvalumab, platinum chemotherapy, and radiation therapy, followed by durvalumab monotherapy for one year. She was assessed as a stable disease without any immunological adverse events. One month after the last ICI injection, she developed pain and swelling in her right SCJ accompanied by fever, resulting in restricted range of arm and neck motion (**Figure 1**). Laboratory blood tests indicated a white blood cell count of 5,100/μL (reference range: 5,000–10,000/uL) and an elevated C-reactive protein (CRP) level of 10.74 mg/dL (normal: < 0.3 mg/dL). Computed tomography (CT) revealed an increased fatty tissue concentration, and magnetic resonance imaging (MRI) showed hyperintensities of short tau inversion recovery (STIR) surrounding the SCJ (**Figures 2A, B**). No signs of bone fracture, abscess, crystal deposition, osteonecrosis, or metastasis were observed. The clinical presentation suggested the possibility of panniculitis, however, given that the patient had been receiving durvalumab, there was also a risk of immune checkpoint inhibitor-induced inflammatory arthritis (ICI-IA). Lacking immediate diagnostic confirmation, the treating physician at the referring hospital implemented a comprehensive treatment strategy, addressing both potential bacterial infections and ICI-IA. She received a combination of ampicillin/sulbactam, garenoxacin, and glucocorticoid therapy, starting with intravenous methylprednisolone 500 mg for one day, followed by oral prednisolone 20 mg/day tapered off in 25 days. Although the patient’s CRP improved to 1.0 mg/dL, arthralgia in the right SCJ persisted with a numerical rating scale (NRS) pain score of 8 under the administration of NSAIDs and a fentanyl patch. Consequently, she was referred to our hospital’s rheumatology department for further assessment and treatment more than a week after the cessation of both antibiotics and glucocorticoid therapy at the referring hospital.

On examination, erythematous swelling, and tenderness in the SCJ persisted, with no evidence of arthritis in any other joints. A totally implantable central venous access port was found in the left subclavian vein without soreness or redness. No plaques or pustulosis were found on her skin, including nails, palms, and soles. Blood analysis showed an erythrocyte sedimentation rate (ESR) of 79 mm/hr (normal: ≤ 15 mm/hr) and CRP of 0.72 mg/L, while other tests, such as rheumatoid factor, anti-cyclic citrullinated peptide antibody, and antinuclear antibodies, were negative. Blood and urine cultures yielded negative results. Ultrasonography-guided joint fluid aspiration was attempted; however, it revealed neither signs of an abscess nor sufficient joint effusions for culture. The subsequent (18) F-fluorodeoxyglucose positron emission tomography (FDG-PET) revealed increased uptake at the right SCJ (SUVmax = 4.1) (**Figure 2C**).

Despite 24 days of antibiotic therapy, there was minimal improvement in the skin manifestations and symptoms, raising doubts about the initial diagnosis of panniculitis. Furthermore, the presence of hyperintensities in STIR-MR imaging and abnormal uptake in FDG-PET at the SCJ indicated that the inflammatory changes were more consistent with inflammatory arthritis than with an infectious abscess.

**Figure 3:**
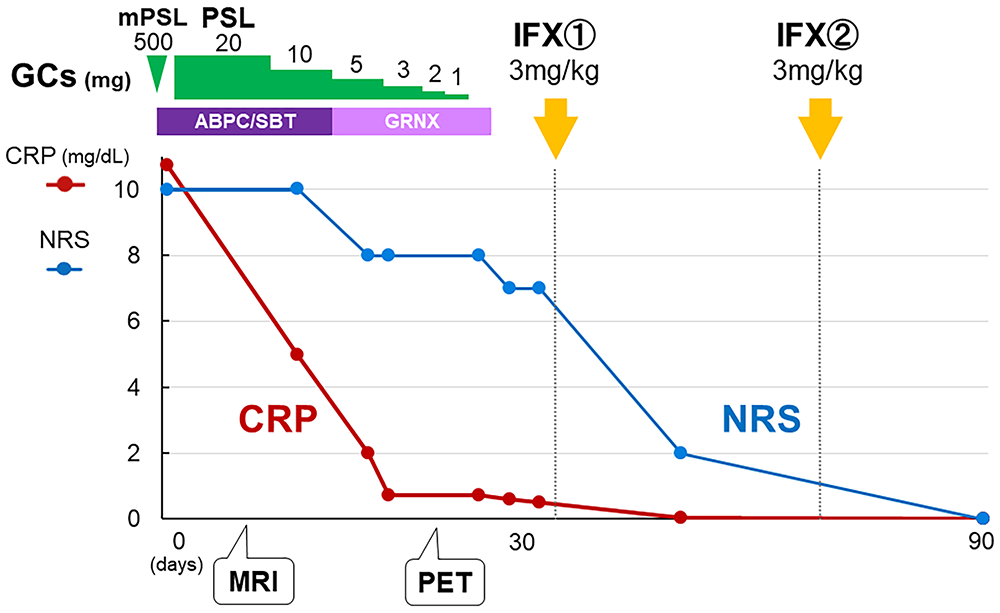
Clinical course. Although the combination of glucocorticoid and antibiotic therapy improved the serum CRP level, the patient’s NRS score remained at 7/10, indicating persistent inflammation. After twice intravenous infliximab induction, NRS score as well as serum CRP level completely resolved. GC: glucocorticoid, mPSL: methylprednisolone, PSL: prednisolone, ABPC/SBT: ampicillin/sulbactam, GRNX: garenoxacin, IFX: infliximab, NRS: numerical rating scale.

The patient was diagnosed with grade 3 ICI-induced right SCJ arthritis (7). Based on the clinical course of resistance to systemic glucocorticoids, she received a 3 mg/kg dose of infliximab intravenously at zero and two weeks. Two weeks after the last injection, all symptoms and serum ESR and CRP levels completely resolved, and her NRS score was 0 (**Figure 3**). Consequently, the patient withdrew from NSAIDs and opioid analgesics.

Apart from the inflammation at the SCJ, the patient did not experience any other irAEs. Although the cancer immunotherapy has been temporarily suspended for a year due to the adverse event, the arthritis has not been recurred and the patient’s condition remains stable.

## Discussion

ICI-IA is one of the rheumatic irAEs and its prevalence may range from 3% to 7.5% (2, 8). Although ICI-IA is known to be a heterogeneous disease, it has been reported that a longer duration of symptoms, use of prescribed glucocorticoids at the first rheumatology visit, and receiving combination ICI therapy are associated with the persistence of ICI-IA (9). To the best of our knowledge, this is the first report of SCJ arthritis occurring during ICI treatment. Differential diagnoses of SCJ swelling can be categorized into five groups: infectious, crystalline, inflammatory, osteoarthritis, and SCJ-specific diseases, including Friedrich disease, condensing osteitis, and SAPHO syndrome (10). Dealing with cancer patients who have a glucocorticoid-resistant clinical course, we often face difficulty in differentiating whether arthritis is indicative of tumor metastasis (11). Although the fluid culture and biopsy could not be obtained from the patient, we ruled out infectious diseases and metastases as much as possible.

SAPHO syndrome is characterized by a combination of skin and osteoarticular manifestations, often involving the SCJ and first sternocostal joints. These may not necessarily coexist; therefore, the absence of skin manifestations does not necessarily exclude the diagnosis of the disease (12). In this case, aseptic inflammation during ICI treatment without evidence of lytic metastases prompted us to suspect that the explanation for SCJ arthritis was the joint involvement of SAPHO syndrome, which is treatable with biological agents.

The treatment of ICI-IAs often relies on knowledge from treatments for rheumatoid arthritis and spondyloarthritis, including methotrexate (MTX), TNF inhibitors (TNFi), and IL-6 receptor inhibitors (IL-6Ri) for preferred options (2, 8). While infliximab has been safely used to treat severe ICI-induced colitis, with most patients resolving symptoms within three months and minimal recurrence risk (13), ICI-IA often necessitates prolonged immunosuppression (2). Therefore, we need careful consideration when administering biological DMARDs (bDMARDs) in the oncologic context. A recent retrospective study with a 9-month follow-up indicates that TNFi do not adversely affect anti-tumor responses (14). This is supported by findings in mouse models that TNFα and other pro-inflammatory cytokines may contribute to tumor microenvironments by promoting tumor-associated macrophages, indicating TNF blockade can reprogram these macrophages towards an antitumor phenotype, potentially enhancing ICI efficacy (15–17). The phase 1b TICIMEL study illustrates that concurrent administration of TNFi with ICIs is safe and may even boost the efficacy of ICIs in treating patients (18). This study, among others, suggests a paradigm shift in how we view the role of inflammation and its modulation in cancer therapy, emphasizing the dual objectives of managing irAEs and maintaining, or potentially augmenting anti-cancer immune responses.

In our case, we opted for TNFi for two reasons. First, according to a recent study (19), bDMARDs allow more rapid arthritis control than MTX, leading to an improvement in a patient’s quality of life. Second, according to its clinical resemblance to SAPHO syndrome, for which there are inadequate data on the efficacy and safety of IL-6Ri (20), TNFi may be favored over IL-6Ri.

In conclusion, ICI-IA with glucocorticoid-resistant progression requires careful exclusion due to the wide range of differential diagnoses; however, moderate-to-severe cases of ICI-IA may benefit from early treatment with bDMARDs to limit the use of glucocorticoids and improve clinical outcomes. Furthermore, infliximab has shown potential therapeutic efficacy in ICI-induced unilateral sternoclavicular arthritis.

## Data availability statement

The original contributions presented in the study are included in the article. Further inquiries can be directed to the corresponding author.

## Ethics statement

Written informed consent was obtained from the individual(s) for the publication of any potentially identifiable images or data included in this article. Written informed consent was obtained from the patient for the publication of this case report.

## Author contributions

SK: Conceptualization, Visualization, Writing – original draft, Writing – review & editing. SS: Conceptualization, Data curation, Supervision, Visualization, Writing – review & editing. HO: Conceptualization, Supervision, Writing – review & editing. AH: Data curation, Supervision, Visualization, Writing – review & editing. KO: Conceptualization, Supervision, Writing – review & editing.
